# Time-to-recovery from severe acute malnutrition in children 6–59 months of age enrolled in the outpatient treatment program in Shebedino, Southern Ethiopia: a prospective cohort study

**DOI:** 10.1186/s12887-019-1407-9

**Published:** 2019-01-28

**Authors:** Genene Teshome, Tafese Bosha, Samson Gebremedhin

**Affiliations:** 1 Regional Health Bureau, Southern Nations, Nationalities and People’s Region, Hawassa city, Ethiopia; 2 School of Nutrition, Food Science and Technology, Hawassa University, Hawassa city, Ethiopia; 3 School of Public Health, Hawassa University, Hawassa city, Ethiopia

**Keywords:** Severe acute malnutrition, Outpatient therapeutic program, Treatment outcome, Time-to-recovery, Diarrhoea, Ethiopia

## Abstract

**Background:**

In Ethiopia uncomplicated severe acute malnutrition (SAM) is managed at health posts level through the outpatient therapeutic program (OTP). Yet, evidence on the treatment success rate of the program is scarce. This study determines the treatment outcomes and predictors of time-to-recovery among children 6–59 months of age with SAM managed at the health posts level in Shebedino district, Southern Ethiopia.

**Methods:**

This was a prospective cohort study that enrolled 216 children with SAM identified through a campaign conducted in May 2015 and treated over eight weeks at 25 health posts of the district. The average time-to-recovery was estimated using Kaplan-Meier survival curve and the independent predictors of the recovery were determined using multivariable Cox-proportional hazard model. The outputs of the analyses are presented via adjusted hazard ratio with 95% confidence intervals (AHR, CI).

**Results:**

At the end of the eight weeks of treatment 79.6% (95% CI: 74.2–85.0%) of cases recovered from SAM with a weight gain rate of 5.4 g/kg/day. The median time-to-recover was 36 days. The analysis indicated, maternal illiteracy (0.54, 0.38–0.78), severe household food insecurity (0.47, 0.28–0.79), walking for more than 1 h to receive the treatment (0.69, 0.50–0.96), diarrhoea co-morbidity (0.63, 0.42–0.91) and practicing sharing of ready to use therapeutic food (RUTF) (0.53, 0.32–0.88) were associated with slower propensity of recovery from SAM. Children who were enrolled with marasmus diagnosis showed lower recovery than children with kwashiorkor (0.30, 0.18–0.51).

**Conclusion:**

The median time-to-recover was 36 days. Discouraging sharing of RUTF, appropriate management of diarrhoea in SAM cases and improving access to OTP sites can help to improve the treatment outcome for SAM.

**Electronic supplementary material:**

The online version of this article (10.1186/s12887-019-1407-9) contains supplementary material, which is available to authorized users.

## Background

Childhood undernutrition remains a major public health problem in the world. Undernutrition is considered as an the underlying cause for nearly half of the global childhood deaths – 3.1 million deaths annually [1, 2]. Despite the significant strides made in promoting child survival in the last two decades, Ethiopia remains one of the countries with highest burden of undernutrition [1, 3]. According to the recent demographic and health survey (DHS), in Ethiopia 38% of the children under the age of five years are stunted; further, 24 and 10% are underweight and wasted, respectively [3].

Severe acute malnutrition (SAM) – the most severe form of malnutrition – is defined as weight-for-height z-score below minus three standard deviations, or middle upper arm circumferences (MUAC) less than 110 mm or the presence of first or second degree bilateral pitting oedema [4]. As of 2016, globally SAM affects 17 million children, of which 98% are either from Asia or Africa [5]. Every year SAM approximately contributes to one million childhood deaths [6]. In Ethiopia, the prevalence of severe wasting is estimated to be 3% [3]. As of 2016, nearly half a million children in the country were in need of treatment for SAM [7].

SAM is a life threatening condition that requires urgent medical attention. The degree of wasting has dose-effect relationship with the risk of death and the risk of mortality is approximately 5–20 folds higher among SAM cases compared to well-nourished children [2]. Furthermore, surviving cases are susceptible to infections and may develop long-lasting physical and cognitive consequences [2].

With timely detection and improved access to standardized treatment, case-fatality rates from SAM can substancially be reduced to less than 5% [8, 9]. Consequently, many countries including Ethiopia have adopted a community- based strategy for scaling up and bringing the treatment closer to the grassroots level [9]. According to the World Health Organization (WHO) and the national guideline, children who have passed an appetite test and are judged to be clinically well should be treated on outpatient bases through the Outpatient Therapeutic Program (OTP). Nevertheless, children with medical complications, severe oedema or poor appetite should be managed as inpatients [4, 10].

In Ethiopia, since 2008 the treatment of uncomplicated SAM had been decentralized to the lowest primary health care unit and shifted to the outpatient setting [10, 11]. Nevertheless, limited information exists regarding the outcome of SAM treatment provided through this decentralized approach. The available few studies employed retrospective design and were reliant on secondary data extracted from medical records [11–16]. Consequently, they might not have captured key variables and can be liable to systematic errors.

Accordingly, this prospective cohort study was conducted to determine level and predictors of time-to-recovery from SAM in children 6–59 months of age managed through the OTP in Shebedino, Southern Ethiopia.

## Methods

### Study setting

The study was conducted from June to August 2015 in Shebedino district of Sidama zone, Southern Ethiopia. The district is located in the Great Rift Valley area, about 300 kms South of Addis Ababa, the capital of Ethiopia. Shebedino is administratively subdivided into 35 kebeles (32 rural and 3 urban). A kebele is the smallest administrative unit in Ethiopia comprising approximately 1000 households. In 2015, Shebedino had an estimated population of 294,214; of these 14% were infants and children 6–59 months of age.

Shebedino is affected by recurrent and chronic food insecurity. In the district, the average farmland ownership by a household is around 0.5 ha. Crop cultivation and livestock rearing are the major livelihood activities in the rural areas. Maize and Enset (false banana) are the major staple foods.

The district has one primary hospital, nine health centers and thirty two health posts, making the potential health service coverage 98%. According to the health care system of Ethiopia, every kebele is expected to have a health post whereby at least two health extension workers (HEWs) are deployed to provide a package of preventive and essential curative services including the management of uncomplicated SAM in children. HEWs identify SAM cases from their catchment area through multiple modalities including periodical growth monitoring and promotion, enhanced outreach strategy (EOS)/community health day (CHD) campaigns, and static service provided at the health post.

### Study design

A prospective cohort study was conducted among children aged 6–59 months with uncomplicated SAM enrolled at the OTP sites of the district following a CHD campaign conducted in late May 2015. The cases were followed for the maximum eight weeks through weekly visits starting from June 01, 2015. However, children who recovered earlier were only followed until recovery. Screening of the children and administration of the treatment were made by the frontline health workers according to the national protocol without any direct involvement of the research team.

### Study participants

All children 6–59 months of age who were newly diagnosed with uncomplicated SAM during the CHD campaign and got enrolled in the OTP program were eligible for the study. According to the national protocol, uncomplicated SAM cases are diagnosed as children with good appetite and no major medical complication having MUAC of less than 110 mm and/or first or second degree bilateral pitting oedema [4].

According to the national protocol patients fulfilling the admission criteria are enrolled and given a weekly Plumpy’Nut ration – trade name of a peanut-based ready-to-use therapeutic food (RUTF). Each week, their weight is taken until they achieve a target weight stated in the protocol. On each visit the children are expected to receive a medical assessment and caregivers should be given nutrition education [4]. As the study employed an observational design, the research team was not involved in any aspect of the treatment of the children.

### Sampling approach

An optimal sample size of 219 children with SAM was determined using Stata 11.0 program based on formula designed for survival analysis. The inputs for the computation were: 95% confidence level, 80% power, 1.5 adjusted hazard ratio to be detected as significant (equivalence of medium effect size) for time-to-recovery outcome variable and 15% compensation for possible non-response. Further, based on the sample size calculation formula for estimating a population average, the sample size (*n* = 219) was considered adequate for determining the median time-to-recovery.

**Fig. 1:**
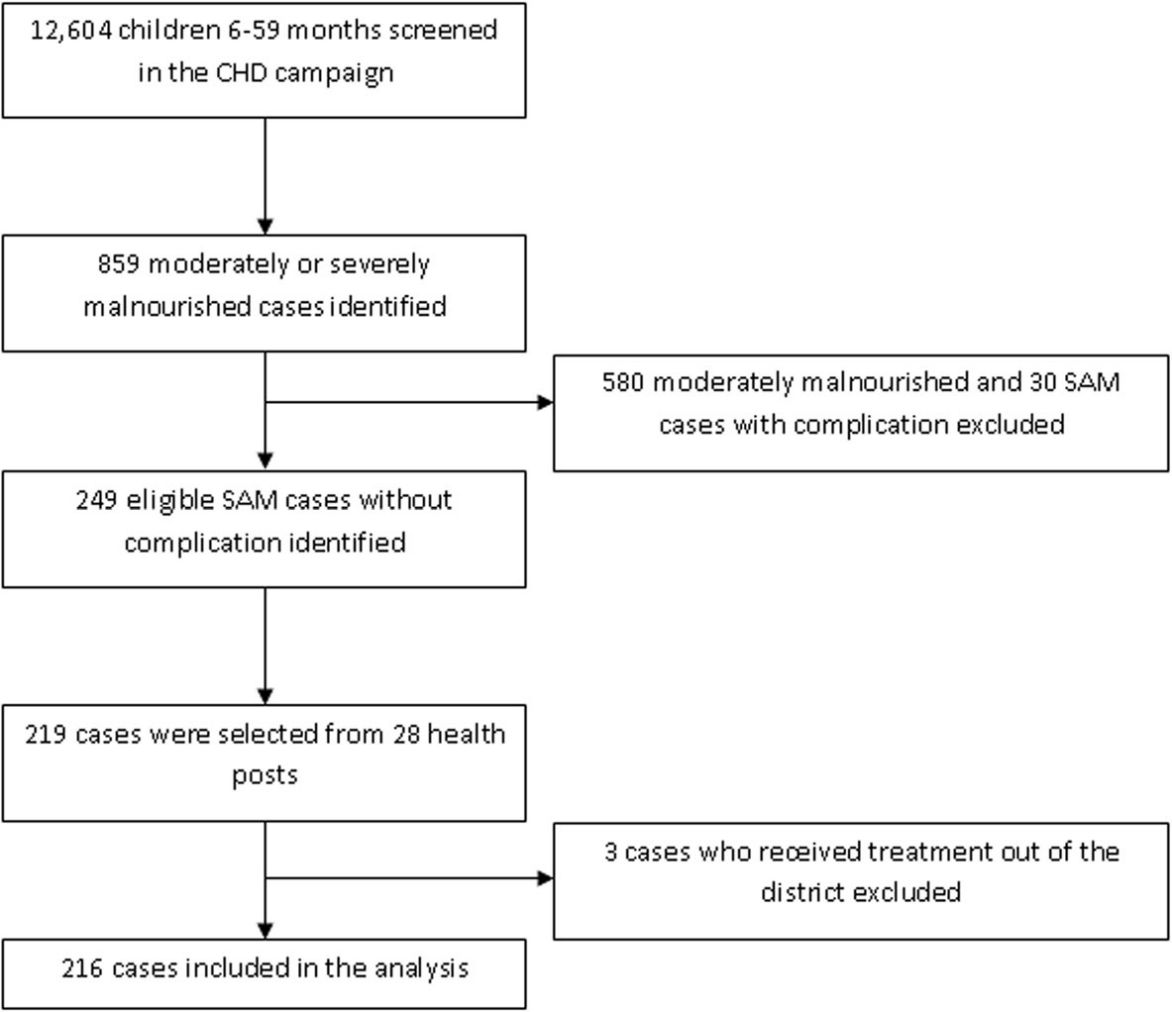
Flowchart of the study

From the total 32 rural health posts found in the district, 25 were selected purposively based on the availability of new SAM cases recruited for OTP during the CHD screening. The total sample size 219 was distributed to health posts proportionally to their newly recruited SAM cases and ultimately the study subjects were selected using quota sampling technique (Fig. 1).

At the end of the CHD camping 219 malnourished children were recruited for the study. Nevertheless, at the first follow-up 3 children were excluded as they were receiving the treatment from health posts found outside Shebedino district. The remaining 216 children were followed for a maximum duration of eight weeks and hence included in the analysis.

### Data collection procedure

Data were gathered by eleven trained enumerators and supervisors using a structured and pretested questionnaire. Baseline data were collected at enrolment and follow-up measurements were made on weekly bases for a maximum of eight weeks.

Socio-demographic and economic variables were gathered at baseline using standard questions extracted from the DHS questionnaire [17]. Dietary Diversity (DD) of the children was assessed at baseline and consecutive weekly follow-up visits by asking the caregivers whether the child had taken from the standard seven food groups recommended by the WHO in the preceding day of the study without setting a minimum intake restriction [18]. The seven food groups were: (i) grains, roots and tubers; (ii) legumes and nuts; (iii) milk and milk products excluding breast milk; (iv) flesh foods; (v) eggs; (vi) vitamin A-rich fruits and vegetables; and (vii) others fruits and vegetables [18]. Household food security was measured at baseline using the Household Food Insecurity Access Scale (HFIAS) by asking about the occurrence and frequency of occurrence of nine food insecurity related events in the preceding four weeks of the survey. Ultimately the food security situation was classified into four ordinal categories: secure, and mild, moderate and severe insecurity [19]. Recent illness history of the child was assessed by asking the caregiver whether the child had fever, cough and diarrhoea in the preceding two weeks of the interview. The questionnaire used for collecting the data is provided as a supporting file with this manuscript (Additional file 1).

Anthropometric measurements – height, weight and MUAC – of the children were taken at baseline and on successive weekly visits using calibrated equipments following standardized procedures. Height and weight were measured without shoes and wearing light clothes using portable stadiometer and Salter spring scales. Height and weight were measured to the nearest 0.1 cm and 100 g, respectively. MUAC was measured at the middle point of the left arm to the nearest 0.1 cm using MUAC tape. Bilateral pitting oedema was assessed by applying normal thumb pressure for 3 s to the both feet.

### Variables of the study

**Fig. 2:**
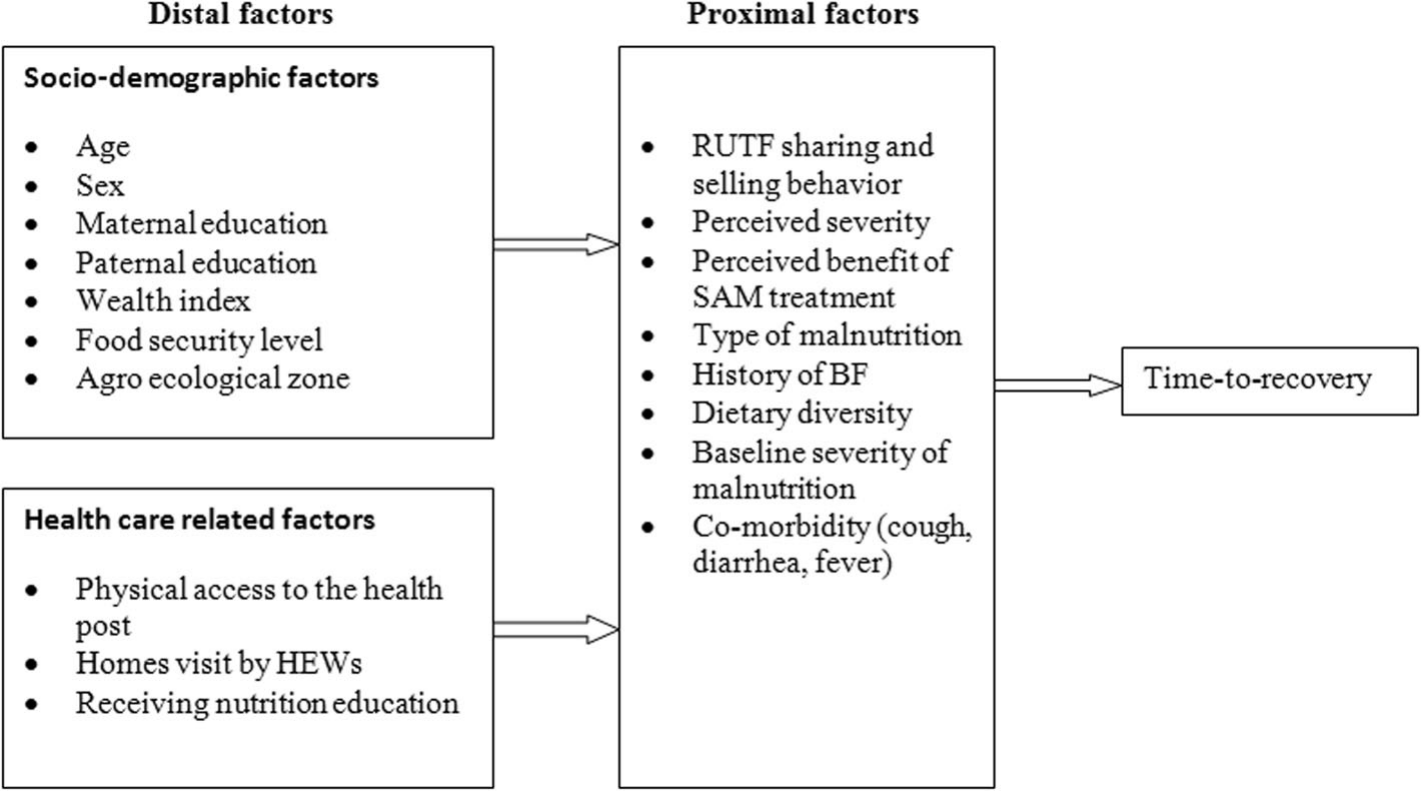
Conceptual framework of the study describing the distal and proximal determinants of time-to-recovery from SAM

The dependent variable of the study is time-to-recover from SAM (i.e. the event of interest is recovery and that the response variable is rate of recovery). The independent variables considered are: age and sex of the child, maternal and paternal educational status, level of household food insecurity, household wealth index, distance from the OTP sites, perceived severity of SAM by the caregivers, perceived benefit of SAM treatment, type of malnutrition (Marasmus or Kwashiorkor), dietary diversity and clinical symptoms (diarrhoea, cough and fever). As described in the following conceptual framework, the independent variables were grouped into distal and proximal factors (Fig. 2).

### Statistical methods

Data were entered, cleaned, and analyzed using SPSS for windows, version 20. Data were described using frequencies, percentages and proper measure of central tendency and dispersion.

During enrollment and follow-ups, dietary diversity scores (DDSs) were determined weekly by summing up the number of unique food groups the child received in the preceding day of the assessment. Ultimately a grand DDS was computed by averaging all the available weekly scores by the number of observations. A grand score of 4 or more was considered as optimal DDS [18].

The treatment outcomes were classified as recovered, non-responder and defaulter in line with the national protocol for the management of SAM [4] and the effectiveness of the program is judged by the Global SPHERE standards [20]. Recovery was defined based on the criteria used to diagnose SAM upon enrollment. For children admitted to OTP based on low MUAC, MUAC greater than 110 mm at two consecutive weeks and/or achieving target weight gain within the maximum stay of 8 weeks in the OTP were used to define recovery. For children admitted based on edema, recovery was resolution of edema at two consecutive weeks. Conversely, children who fail to achieve the aforementioned recovery criteria within the maximum eight weeks treatment were considered as non-responders. Children who missed appointments for two consecutive weeks while being confirmed that they are alive were considered as defaulters.

The time-to-recover from SAM was determined by calculating the differences (in day) from the start of treatment until the child were declared recovered. The average time-to-recover in days was estimated using Kaplan-Meier survival analysis.

Predictors of time-to-recovery were identified using bivariable and multivariable Cox-proportional hazard models (CPHM). All independent variables that had *p*-value less than 0.25 in bivariable model were considered as candidate variables for the multivariable model. In order to avoid over adjustment bias, proximal and distal variables were fitted in separate models in accordance with the conceptual framework of the study. The output of the multivariable CPHM is presented using adjusted hazard ratios (AHR) with the respective 95% confidence intervals (CI). The proportional hazard assumption of the model was assessed on the basis of Schoenfeld residuals. Multicolinearity was checked using variance inflation factor.

For the distal CPHM model a total of eight variables were considered. These were: sex and age of the index child, maternal and paternal educational status, agro-ecology of the kebele, household food insecurity status, household wealth index, two-way travelling distance to the health post, and home visit by HEWs during the follow-up period. In the bivariable analyses, five variables (age of the child, maternal literacy, agro-ecological zone, food insecurity and distance to health post) had *p*-values less than 0.25 and hence considered for the multivariable model.

For the proximal CPHM a total of nine variables were considered. The variables were DDS, type of nutritional diagnosis at baseline, occurrence of diarrhoea, fever and cough, RUTF sharing and selling practices, breastfeeding status and maternal perception on severity of SAM. After the bivariable analyses, all of the variables except breastfeeding status were found eligible (*p*-value < 0.25) for the multivariable analysis.

Household wealth index was computed using Principal Component Analysis (PCA) as an indicator of household wealth status. A total of fifteen variables related to ownership of selected household assets, size of agricultural land, quantity of livestock, materials used for housing construction, and ownership of improved water and sanitation facilities were considered. Ultimately the generated score was divided into quintiles: poorest, poorer, middle, richer, and richest.

### Ethical considerations

The research protocol was reviewed and approved by the institutional review board (IRB) of College of Medicine and Health Science, Hawassa University. Data were collected after securing informed verbal consent from the caregivers of the children. Verbal consent, instead of written consent, was preferred because most of the study respondents were not literate. The same was approved by the IRB that reviewed the protocol of the study. Confidentiality was maintained while handling participants’ information. Nutrition education was given to the entire caregivers.

## Results

### Socio-demographic characteristics

Among 216 study subjects enrolled in the study, the boys-to-girls ratio was 1.08 and at enrolment 36.1% were younger than 24 months of age. The mean (±SD) age of the caregivers was 30.1 (±7.0) years and 87.5% were married. More than two-thirds (72.2%) didn’t attend any formal education and about three-fourths (76.4%) were housewives. Nearly two-thirds (65.7%) of the children were sampled from midland areas (1750 to 2300 m above sea level) (Table 1).

**Table 1:** Socio-demographic and economic characteristics of the study participants

Variables (*n* = 216)	Frequency	Percent
Sex of the child		
Male	104	48.1
Female	112	51.9
Age of children (months)		
6–11	44	20.4
12–23	34	15.7
24–35	29	13.4
36–47	46	21.3
48–59	63	29.2
Maternal education		
No formal education	156	72.8
Primary school	60	27.2
Paternal education		
No formal education	151	69.9
Primary school	65	30.1
Mother’s occupation		
Housewife	165	76.4
Others	51	23.6
Agro-ecological zone		
Highland	74	34.3
Midland	142	65.7

### Nutritional and related characteristics of children at OTP enrollment

Household food insecurity assessment at baseline indicated that all of the households had experienced food insecurity with different degrees of severity in the preceding four weeks of the survey. Nearly half of the respondents (45.4%) had to walk for more than an hour to receive the OTP service from the nearby health post.

At baseline 68.1% of the cases were Marasmic (MUAC< 110 mm) while the remaining 31.9% had Kwashiorkor (presence of bilateral pitting oedema irrespective of anthropometric status). Study participants enrolled in the study with an average weight (±SD) of 8.5 (±2.6) kgs. On admission the vast majority (91.2%) of the children had suboptimal DDS. But nearly half (47.7%) of them were still breastfeeding. Regarding the occurrence of common childhood ailments, 12.5, 42.4 and 45.1% of the children, had cough, diarrhea and fever in the reference two weeks, respectively.

During the first follow-up visit carried out a week after OTP enrolment, the RUTF utilization pattern was assessed. It was found that RUTF sharing (35.2%) and selling (20.8%) practices were not rare. Nearly quarters (24.5%) of the respondents were aware that RUTF is both food and medicine to children with severe malnutrition (Table 2).

**Table 2:** Nutritional and related characteristics of children with SAM enrolled in OTP

Variables (*n* = 216)	Frequency	Percent
Household food security status		
Secure	0	0.0
Mild	22	10.2
Moderate	53	24.5
Severe	141	65.3
Two-way walking distance to the OTP		
Less than an hour	118	54.6
More than an hour	98	45.4
Visited at home by HEWs during the treatment		
Yes	67	31.0
No	149	69.0
Receiving nutrition education during the treatment		
Yes	147	68.1
No	69	31.9
Nutritional diagnosis at admission		
Marasmus	147	68.1
Kwashiorkor	69	31.9
Ailment in the past 2 weeks		
Diarrhoea	115	42.4
Cough	34	12.5
Fever	122	45.1
Breastfeeding status at admission		
Still breastfeeding	103	47.7
Stopped breastfeeding	113	52.3
Dietary diversity score at admission		
Suboptimal (< 4)	197	91.2
Optimal (≥4)	19	8.8
RUTF sharing at first follow-up		
Yes	76	35.2
No	140	64.8
RUTF selling during the first week		
Yes	45	20.8
No	171	79.2
Caregivers perception on RUTF		
Food and medicine	87	40.3
Food for SAM child	53	24.5
Medicine for SAM child	76	35.2

### Time-to-recovery and treatment outcomes of children with SAM

From the total study subjects, 79.6% (95% CI: 74.2–85.0%) successfully recovered from SAM within the first eight weeks of treatment. Conversely, nearly one-fifth (20.4%) were censored. Reasons for censoring were: failure to respond to the treatment (11.1%), defaulting from the treatment (3.7%) and transferred out (5.6%).

The median time-to-recovery as determined by the Kaplan-Meier survival analysis, was 5 weeks (95% CI: 4.67–5.33) or 36.0 days (95% CI: 34.3–37.7). The overall mean (±SD) daily weight gain rate was 5.4 (2.6) gm/kg/day for the recovered children.

### Determinants of recovery from SAM

Predictors of recovery were identified using Cox-proportional hazard model fitted separately to the distal and proximal factors in line with the conceptual framework of the study. In the distal multivariable model, maternal education status, agro-ecological zone of the kebele, household food insecurity status, and distance from the OTP site turned out to be significant predictors of recovery from SAM. Children having caregivers with no formal education had 46% reduced chance of recovery than their counterparts. Children from the highlands showed 43% lower probability of recovery as compared to those from the midlands. Those from severely food insecure household were 53% less likely to recover than cases from mildly food insecure households. Children who reside more than an hour walking distance from the OTP site had 31% reduced chance of recovery than their counterparts (Table 3).

**Table 3:** Outputs of the Cox-proportional hazard model analyses on the distal and proximate predictors of time-to-recovery from severe acute malnutrition

Independent variables (*n* = 216)	CHR (95% CI)	AHR (95% CI)
Age group of child		
Younger than 24 months	1^r^	1^r^
Older than 24 months	1.58 (1.47–2.17)*	1.17 (0.82–1.66)
Sex of child		
Male	1^r^	–
Female	0.86 (0.89–1.18)	–
Maternal education		
Primary school	1^r^	1^r^
No education	0.51 (0.37–0.72)*	0.54 (0.38–0.78)*
Paternal education		
Primary school	1^r^	–
No education	0.34 (0.95–1.88)	–
Ecological zone		
Midland	1^r^	1^r^
Highland	0.62 (0.44–0.86)*	0.57 (0.41–0.81)*
Household food security status		
Mild insecurity	1^r^	1^r^
Moderate insecurity	0.77 (0.45–1.32)	0.68 (0.39–1.71)
Severe insecurity	0.43 (0.26–.71)*	0.47(0.28–0.79)*
Household wealth index		
Richest	1^r^	
Richer	0.92 (0.56–1.51)	–
Middle	0.89 (0.54–1.47)	–
Poorer	1.02 (0.63–1.67)	–
Poorest	0.80 (0.50–1.32)	–
Two-way distance from health post		
More than an hour	0.59(0.43–0.81)*	0.69 (0.50–0.96)*
Less than an hour	1^r^	1^r^
Dietary diversity score		
Optimal	1^r^	–
Suboptimal	0.86 (0.50–1.50)	–
Nutritional diagnosis at admission		
Marasmus	0.18 (0.12–0.27)*	0.30 (0.18–0.51)*
Kwashiorkor	1^r^	1^r^
Diarrhea during admission or follow-up		
Yes	0.40 (0.29–0.55)*	0.63 (0.42–0.91)*
No	1^r^	1^r^
Cough during admission or follow-up		
Yes	1^r^	1^r^
No	0.55 (0.35–0.86)*	0.65 (0.41–1.03)
Fever during admission or follow-up		
Yes	1^r^	–
No	0.90 (0.66–1.21)	–
RUTF sharing practice		
Yes	0.56 (0.40–0.77)*	0.53 (0.32–0.88)*
No	1^r^	1^r^
RUTF selling practice		
Yes	0.56 (0.38–0.85)*	0.61 (0.29–8.33)
No	1^r^	1^r^
Maternal perceived on the severity of SAM		
Not aware of any consequences	1^r^	1^r^
Death and disability	1.42 (0.82–2.49)	1.17 (0.66–2.08)
Disability	1.11(0.63–1.55)	1.03 (0.58–1.84)
Death	4.10 (2.32–6.87)*	2.45 (1.35–4.46)*

* Significant association at *p*-value of 0.05

1^r^ Set as a reference group

*CHR* crude hazard ratio, *AHR* adjusted hazard ratio, *CI* confidence interval

In the proximate model six variables emerged statistically significant. SAM cases who were admitted on the basis of low MUAC were less likely to recover than those admitted based on presence of edema. Children who had diarrhoea at baseline or during follow-up had 37% reduced probability of recovery than their counterparts. The chance of recovery was almost reduced by half among children whose RUTF was shared with other household members. Furthermore, among children whose caregivers were aware that SAM can be fatal, the chance of recovery was nearly two times higher (Table 3).

## Discussion

This study assessed the recovery rate of severely malnourished infants and children aged 6–59 months managed on outpatient basis for a maximum duration of eight weeks. The recovery rate was about 80%. Time-to-recovery was negatively affected by manifold factors including maternal illiteracy, severe household food insecurity, inaccessibility of OTP sites, diarrhoea co-morbidity, practice of RUTF sharing within the household and being diagnosed with Marasmus on admission.

The level of recovery reported in this study is above the minimum 75% threshold set by the SPHERE standard [20]. Previous studies in Ethiopia that evaluated the recovery rate in the OTP program provided at health center and/or health post levels came up with assorted figures. Studies that evaluated OTP provided at health post level in North Western Ethiopia and Wolita determined 78 and 65% recovery rates, respectively [11, 20]. Studies in Jimma (45%), South Wollo (82%) and Southern Ethiopia (87%) that evaluated health center level OTP care reported varying recovery rates [12, 16, 21]. Studies in Tigray region (62%) and Kemba district (68%) based on combination of cases treated at health center and health post levels reported relatively lower success rates [14, 15]. The observed discrepancies could be due to diverse reasons including variation in timing and season in which the studies were conducted, level of maturity of the OTP program in the study settings and dissimilarity in the underlying determinants of malnutrition across the localities.

The mean weight gain rate of 5.4 g/kg/day observed was less than the expected rate based on the SPHERE standard which recommends weight gain rate greater than 8 g/kg/day [20]. Many studies conducted in Ethiopia [11, 13, 21] and in East Africa [22, 23] consistently documented substandard rate of weight gain among SAM cases managed through the OTP. A study in Southern Ethiopia found 4.5 and 3.5 g/kg/day weight gain in Kwashiorkor and Marasmic cases, respectively [11]. Another study from Wolaita zone, Southern Ethiopia determined 4.2 g/kg/day rate [13].

Overall the median time-to-recovery was about 5 weeks (36 days). It is within the range of the acceptable minimum international standard (< 6 weeks) [24] and it is well within the Ethiopian protocol for management of SAM which allows children to stay under treatment up to 8 weeks [4, 8]. Previous studies in Ethiopia reported comparable figures. In a study based on OTP care provided at health post level in Wolita zone, the time to recovery was 35 days for children with kwashiorkor and 49 days for children with marasmus [13]. A similar study North Western Ethiopia reported 48 days [11]. According to a study in Jimma that evaluated health center level OTP care, the median time to recovery was 38 days [16]. In a similar study in Southern Ethiopia the time ranged from 21to 25 days depending on the type of malnutrition [12]. In studies conducted in Tigray region and Kemba district the time to recovery was approximately 49 days [14, 15].

In the current study, maternal literacy is identified as a significant predictor of recovery of children from SAM. Previous studies which were based on secondary medical records review have not explored such relationship as socio-demographic information is not registered in the standard OTP cards. However, the finding is plausible and anticipatable as maternal literacy is likely to be associated with better child feeding and caring practice, adoption of nutritional advices and superior household economic status.

The study found that children from severely food insecure households showed lesser propensity of recovery from SAM. Better household food security level is likely to promote the recovery of children through enabling caregivers to adhere to the nutritional advices provided by health workers. Household food insecurity may also prompt mothers to share RUTF with other members of the household.

Conversely, the study did not witness significant association between household wealth index and time-to-recovery from SAM. The unexpected finding can be due to the fact that wealth was quantified using a relative scale as measurement based on actual household income was not feasible. Relative scale might not have adequate discriminating power to disaggregate a population with homogeneous economic status. The adjustment of household food insecurity for household wealth index might have also caused underestimation of the association as the two variables are likely to be correlated to each other.

In the study area the majority of the caregivers travelled for less than an hour walking distance to receive the OTP service. The finding is compatible with the standard of CMAM programs which aims to provide services within 3 h walking distance [20]. However, significantly lower time-to-recovery rate was observed among caregivers who travel more than one hour to receive the service. Caregivers who have limited access to OTP sites may only decide to bring their child to treatment when the malnutrition gets severe and this may compromise the treatment success rate. The finding may also indicate that making the OTP service even more accessible to the community may help to improve the treatment outcome.

Cases with oedematous malnutrition demonstrated a better propensity of recovery than severely wasted children. This is parallel to the findings of the two studies conducted in Ethiopia [11, 12]. A study from North Western Ethiopia concluded that the median time-to-recovery was 35 days for children with Kwashiorkor and 49 days for children with Marasmus [11]. While the study in Southern Ethiopia reported 25 and 21 days average length of stay in the treatment respectively for the two groups [12]. A study that evaluated the outcome of inpatient SAM cases concluded the same [25]. The observed variation can likely be due to differences in the severity of wasting between the two groups on enrolment. Further, Kwash cases, unlike Marasmic children, are discharged from the OTP upon the resolution of oedema regardless of their weight gain progress [15].

We observed that diarrhoea complicates SAM almost in two-fifth of the children. Further, diarrhoea while on treatment is a negative predictor of time-to-recovery from SAM. Diarrhoea is known to be more frequent in SAM cases due to the systematic immune-suppression effect and loss of the intestinal mucosal barrier due to malnutrition [26]. A study conducted in Tigray Northern Ethiopia also found slower recovery rates among children who had diarrhoea during the course of SAM [15]. Diarrhoea may retard weight gain during treatment through compromising absorption and increasing biological demand for nutrients. Other co-symptoms like anorexia and vomiting may also limit recovery from SAM.

Nearly one-in-three of the caregivers with SAM children reported the practice of sharing RUTF with other members of the household. Even the figure is likely to be underestimated due to social desirability bias. Our study also found that the practice as a significant negative predictor of time-to-recovery from SAM. Previous studies have also concluded the same [16, 24, 27, 28].

The findings of the study have to be interpreted inconsideration of its strength and limitation. Unlike most of the earlier studies that evaluated OTP programs, our study employed a prospective cohort design and used primary data. Accordingly we have been able to evaluate the significance of many socio-demographic, economic and nutritional factors which are otherwise unavailable in medical records.

Conversely, we could have underestimated the significance of the OTP program as the study was conducted during the lean season of the locality. As dietary diversity and household food insecurity were assessed retrospectively, recall errors cannot be fully excluded. Further, responses related to RUTF sharing and selling behaviours might have been underestimated due to social desirability bias. In addition, baseline wasting status, which can be an important predictor of the treatment outcome in children with non-oedematous malnutrition was not statistically adjusted, and this may have limited the comprehensiveness of the model and caused residual bias in the analysis. Due to the observational design of the study, confounding from unmeasured variables (e.g. vaccination history, birth weight) cannot be entirely excluded.

## Conclusion

The OTP program in Shebedino exceeds the international minimum standard for recovery in most of the indicators. The median time-to-recover from SAM was 36 days. Factors that prolong time-to-recovery include maternal illiteracy, severe household food insecurity, practice of RUTF sharing within the household, lack of access to the OTP sites, being Marasmic at enrolment and diarrhoea co-morbidity during admission or follow-up.

The CMAM program in the district or in other similar settings can enhance treatment outcome by improving access to OTP sites, discouraging of RUTF sharing behaviour and giving close follow-up to children with diarrhoea co-morbidity.

## Additional file

Additional file 1: Questionnaire used for data collection. (DOCX 31 kb)

## Abbreviations

AHR: Adjusted Hazard Ratio
CHD: Community Health Day
CI: Confidence Intervals
CPHM: Cox Proportional Hazard Model
DD: Dietary Diversity
DHS: Demographic and Health Survey
EOS: Enhanced Outreach Strategy
HEWs: Health Extension Workers
HFIAS: Household Food Insecurity Access Scale
IQR: Inter Quartile Range
IRB: Institutional Review Board
MUAC: Middle-upper Arm Circumference
OTP: Outpatient Therapeutic Program
RUTF: Ready-to-use Therapeutic Food
SAM: Severe Acute Malnutrition
SD: Standard Deviation
SPSS: Statistical Package for Social Sciences
WHO: World Health Organization
