# Supplementary material for: Time-to-recovery from severe acute malnutrition in children 6–59 months of age enrolled in the outpatient treatment program in Shebedino, Southern Ethiopia: a prospective cohort study
Source: BMC Pediatr. 2019 Jan 28;19:33. doi: 10.1186/s12887-019-1407-9 (PMC6348627; doi:10.1186/s12887-019-1407-9)
Supplement: Supplementary file 1 — Questionnaire used for data collection. (DOCX 31 kb) [file 12887_2019_1407_MOESM1_ESM.docx]

## Questionnaire (English version)

**Participant’s code: ___________**

**Identification**

1. Time interview: started_____________ completed _______________
2. House number _____________________________________
3. Date D/M/Y of Interview _____________________________________
4. Name of Interviewer _____________________________________
5. Supervised & checked by _____________________________________
6. **BASELINE QUESTIONNAIRE**

## Section-1.1: Socio-demographic characteristics

| S.No | Questions | Responses | Skip |
| --- | --- | --- | --- |
| 1 | Age respondent?( In completed years) | ________years |  |
| 2 | What is your relationship to the child? | 1.Biological mother  2.Grandmother  3.Sister  4.Stepmother  5.Otherfemale relatives  6.Brother 7  7.Father  Other (specify)__________ |  |
| 3 | Age of the child (In completed month) | ________month |  |
| 4 | Sex of the child | 1.Male  2.Femal |  |
| 5 | Birth order of the child | 1.First child  2.Second child  3.Third child  4.Forth child  5.Fifth child and above  Other (specify |  |
| 6 | What is your marital Status? | 1.Singl  2.Married/living together  3.Divorced/separated  4.Widowed |  |
| 7 | What is your current educational status | 1.Illiterate  2.Readorwrite  3.Formal education (Grade_)  4.Collage level and abo4 |  |
| 8 | What is educational status of child’s father? | 1.Illiterate  2.Readorwrite  3.Formal education (Grade_)  4.Collage level and abo4 |  |
| 9 | What is your religion |  |  |
| 10 | What is your ethnicity? | 1.Sidama  2.Amhara  3.Oromo  4.Wolait  5.Gedeo  Other (Specify)______ |  |
| 11 | What is your main  Occupational status? | 1.Housewife  2.Merchant  3.Civil servant  4.Daily laborer  5.Farmer  6.Student  Others (specify)________ |  |
| 12 | Family/ household head | 1.Mother  2.Father  3.Someone else |  |
| 13 | Age of the household head  (in completed years) | _______years |  |
| 14 | How many people usually live in this household? | Number of people_____ |  |
| 15 | How many children of age6-59months usually live in this household? | Number children______ |  |
| 16 | Is the family registered for general food ration (GFR)? | 1.Yes  2.No |  |
| 17 | Is the child breastfeeding? | 1.Yes  2.No |  |

**Section-1.2 Household wealth status**

| **S.no** | **Questions** | **Responses** | **Skip** |
| --- | --- | --- | --- |
| 18 | Household ownership? | 1.Owned  2.Rented  3.Dependent  Other (specify)___________ |  |
| 19 | What is the main source of drinking water for members of your household? | **Piped water**  1. Piped into dwelling  2.Piped to yard/plot  3.Public tap/standpipe  4.Borehole  **Dug well**  5.Protected well  6.Unprotected well  **water from spring**  7.Protected spring  8.Unprotected spring  9.Rainwater  **Surface water**  10.River/lake/pond/stream/dam  Other (Specify)_________ |  |
| 20 | which type of toilet your household members use? | 1.Flush toilet  2.Traditional Pit latrine  3.Composting toilet  4.Bush or field  5.Ongroundwithin compound  Other (specify)____________ |  |
| 21 | Does your household have:  A watch/clock?  A radio?  A television?  A mobile telephone?  A non-mobile telephone?  A table?  A chair?  A bed with cotton/sponge mattress? | Yes No  A sewing machine.................1 2  A watch/clock ………….......1 2  A radio………………….. 1 2  A television.................... 1 2  A mobile telephone......... 1 2  A non-mobile telephone….. 1 2  A table………………….. 1 2  A chair………………….. 1 2  A bed with cotton  sponge mattres……………… 1 2 |  |
| 23 | Main material of the floor. | 1.Earth/Sand  2.Dung  3.Wood planks  4.Palm/bam  5.Wood  6.Cement  Other (specify)__________ |  |
| 24 | Main material of the roof? Obseve | 1.No roof 2. Thatch/leaf/mud  3.Corrugated iron /metal/Wood  Other (specify)____________ |  |
| 25 | How many animals of each type do you have? | Cows__ Oxen___  Sheep__ Goats ___  Horses___Donkeys___Mule___  Chicken______ |  |
| 26 | Do you have your own agricultural land? | 1.Yes  2.No |  |
| 26 | If yes, to Q #26, What is the size of your land? Note: 1 ‘*Timad*’= ¼ ha | Timad______  Hectar_______  Other Specify_________ |  |

**Section-1.3 Factors associated with recovery**

| 27 | What type of crop produce on your land?  (multiple answer possible) | 1. Legume  2. Grains/cereals  3. Enset  4.Cash crops  Other (specify) |  |
| --- | --- | --- | --- |
| 28 | What is agro-ecological zone of the area? Or altitude of the area? | 1.Lowland  2.Midland  3.Highland |  |
| 29 | Distance of OTP sites from the child home | _________minute |  |
| 30 | Do you know that this severe acute malnutrition can kill your child? | 1.Yes  2.No |  |
| 31 | Do you know that this severe acute malnutrition can exposes your child to different illness? | 1.Yes  2.No |  |
| 32 | Again do you have any information that severe acute malnutrition can causes long term?  (Multiple answer possible) | 1.Mental retardation  2.Social problem  3.Psychological problem  4.Poor School performance |  |
| 33 | Who has more control over all the household’s food basket or resources? | 1.Wife  2.Husband  3.Someone else |  |
| 34 | From the following in what ways your husband supports you? Multiple answer possible | 1.Feeding child  2.Washing child  3.Giving money  4.Bringing plumpy’nut  5.Taking child to OTP  Other specify____________ |  |

**1.4 Household food Security level questions**

| NO | QUESTION | RESPONSE | skip |
| --- | --- | --- | --- |
| 35 | In the past four weeks, did you worry that your household would not have enough food? | 0 = No (skip to Q2)  1=Yes |  |
| 35.a | How often did this happen? | 1 = Rarely (once or twice in the past four weeks)  2 = Sometimes (three to ten times in the past four weeks)  3 = Often (more than ten times in the past four weeks) |  |
| 36 | In the past four weeks, were you or any household member not able to eat the kinds of foods you preferred because of a lack of resources? | 0=No (skip to Q3)  1=Yes |  |
| 36.a | How often did this happen? | 1 = Rarely (once or twice in the past four weeks)  2 = Sometimes (three to ten times in the past four weeks)  3 = Often (more than ten times in the past four weeks) |  |
| 37 | In the past four weeks, did you or any household member have to eat a limited variety of foods due to a lack of resources? | 0 = No (skip to Q4)  1 = Yes |  |
| 37.a | How often did this happen? | 1 = Rarely (once or twice in the past four weeks)  2 = Sometimes (three to ten times in the past four weeks)  3 = Often (more than ten times in the past four weeks) |  |
| 38 | In the past four weeks, did you or any household member have to eat some foods that you really did not want to eat because of a lack of resources to obtain other types of food? | 0 = No (skip to Q5)  1 = Yes |  |
| 38.a | How often did this happen? | 1 = Rarely (once or twice in the past four weeks)  2 = Sometimes (three to ten times in the past four weeks)  3 = Often (more than ten times in the past four weeks) |  |
| 39 | In the past four weeks, did you or any household member have to eat a smaller meal than you felt you needed because there was not enough food? | 0 = No (skip to Q6)  1 = Yes |  |
| 39.a | How often did this happen? | 1 = Rarely (once or twice in the past four weeks)  2 = Sometimes (three to ten times in the past four weeks)  3 = Often (more than ten times in the past four weeks) |  |
| 40 | In the past four weeks, did you or any other household member have to eat fewer meals in a day because there was not enough food? | 0 = No (skip to Q7)  1 = Yes |  |
| 40.a | How often did this happen? | 1 = Rarely (once or twice in the past four weeks)  2 = Sometimes (three to ten times in the past four weeks)  3 = Often (more than ten times in the past four weeks) |  |
| 41 | In the past four weeks, was there ever no food to eat of any kind in your household because of lack of resources to get food? | 0 = No (skip to Q8)  1 = Yes |  |
| 41.a | How often did this happen? | 1 = Rarely (once or twice in the past four weeks)  2 = Sometimes (three to ten times in the past four weeks)  3 = Often (more than ten times in the past four weeks) |  |
| 42 | In the past four weeks, did you or any household member go to sleep at night hungry because there was not enough food? | 0 = No (skip to Q9)  1 = Yes |  |
| 42.a | How often did this happen? | 1 = Rarely (once or twice in the past four weeks)  2 = Sometimes (three to ten times in the past four weeks)  3 = Often (more than ten times in the past four weeks) |  |
| 43 | In the past four weeks, did you or any household member go a whole day and night without eating anything because there was not enough food? | 0 = No (questionnaire is finished)  1 = Yes |  |
| 43.a | How often did this happen? | 1 = Rarely (once or twice in the past four weeks)  2 = Sometimes (three to ten times in the past four weeks)  3 = Often (more than ten times in the past four weeks) |  |

#

# 2. Follow up questionnaire

**Section-2.1 Dietary Diversity Question**

**45.** Since this time yesterday has the child received any of the following? Yes=1 No=2

| A | Any food made from cereals such as teff, sorghum, wheat, maize and barley | 1 | 2 |
| --- | --- | --- | --- |
| B | Any food made from legumes (lentils, beans, soybeans, pulses, peas, linseed sesame) and nuts | 1 | 2 |
| C | Dairy products (milk, yogurt, cheese)and meat( liver/organ meats), eggs, poultry, fish | 1 | 2 |
| D | Any food made from tubers or roots, such as white potatoes, local roots/ tubers, onion | 1 | 2 |
| E | Any food made from carrots, red sweet potatoes, and green leafy vegetables(Vitamin-A rich fruits and vegetables) | 1 | 2 |
| F | Any other fruits/ vegetables such as orange, lemon, banana, papaya | 1 | 2 |
| G | Any food made with oil or butter | 1 | 2 |
| H | Other (specify)………………………………………………………. |  |  |

**2.2 Illnesses history of the child during recent two weeks**

| 46 | Try to remember the last two weeks. During this period, has the child had diarrhea? | 1.Yes  2.No  Others________ |  |
| --- | --- | --- | --- |
| 47 | Try to remember the last two weeks again. Has the child had a cough and difficulty breathing? | 1.Yes  2.No  Others________ |  |
| 48 | Try to remember the last two weeks again, has the child had fever? | 1.Yes  2.No  Others________ |  |
| 49 | Does your child share the Plumpy nut with other siblings or children or members of the family in the compound? (starting from enrollment into the program) | 1.Yes  2.No  Others________ |  |
| 50 | Do know that Plumpy nut selling is a common practice in your village? Have you ever bought or sold it? | 1.Yes  2.No  Others________ |  |
| 51 | In your view, what is the main purpose of feeding child the Plumpy nut you got from the OTP? | 1.To cure the child  2.For normal growth  3.Don’t know  Other (specify)____ |  |
| 52 | Do you think that this Plumpy nut can cure your child? | 1.Yes  2.No |  |
| 53 | Have you ever received any piece of advice specifically with regard to Nutrition education message about Plumpy nut? | 1.Yes  2.No |  |
| 54 | In the last15 days have you received any education/counseling regarding child feeding? | 1.Yes  2.No |  |
| 55 | In the last 15 days was there any one who visits the child at your home? | 1.Yes  2.No |  |

**2.3 Weekly assessment**

| WEEK | DATE  (dd/mm/yy) | HEIGHT  (cm) | WEIGHT  (kg) | MUAC  (cm) | EDEMA  (+,++,) | HOME VISIT | |
| --- | --- | --- | --- | --- | --- | --- | --- |
|  |  |  |  |  |  | Date | Findings |
| 1 |  |  |  |  |  |  |  |
| 2 |  |  |  |  |  |  |  |
| 3 |  |  |  |  |  |  |  |
| 4 |  |  |  |  |  |  |  |
| 5 |  |  |  |  |  |  |  |
| 6 |  |  |  |  |  |  |  |
| 7 |  |  |  |  |  |  |  |
| 8 |  |  |  |  |  |  |  |
|  | **Outcomes charts** | |  | | |  |  |
| 57 | Weight gain | | Write in g/kg/day____ | | |  |  |
| 58 | Length of stay | | Write in days______ | | |  |  |
| 59 | Treatment outcome | | 1.Recovered/cured  2.Died  3.Defaulted  4.Non-responder | | |  |  |
